# Functional antibody signatures following therapeutic immunization in Simian and Human immunodeficiency virus infection

**DOI:** 10.1038/s41541-026-01390-3

**Published:** 2026-01-31

**Authors:** Shlomi Ilan, Yannic Bartsch, Wonyeong Jung, Evgenii Kliuchnikov, Vicky Roy, Riley Bonifer, Victoria E. K. Walker-Sperling, Erica N. Borducchi, Joseph P. Nkolola, Douglas A. Lauffenburger, Daniel J. Stieh, Dan H. Barouch, Boris Julg

**Affiliations:** 1 Ragon Institute of Mass General, MIT and Harvard, Cambridge, MA USA; 2 Laboratory of Anti-Viral Antibody-Omics, TWINCORE-Institute for Experimental Infection Research, Helmholtz Center for Infection Research (HZI) and Medical School Hannover (MHH) and Cluster of Excellence RESIST (EXC 2155), Hannover, Germany; 3 Department of Biological Engineering, Massachusetts Institute of Technology, Cambridge, MA USA; 4 Center for Virology and Vaccine Research, Beth Israel Deaconess Medical Center, Boston, MA USA; 5 Janssen Vaccines & Prevention B.V., a Johnson & Johnson companies, Leiden, Netherlands

**Keywords:** Diseases, Immunology, Microbiology

## Abstract

Reducing the latent HIV-1 reservoir is essential to achieving a functional cure, and therapeutic vaccination is a promising strategy. While most approaches emphasize cytotoxic CD8⁺ T-cell responses, the role of antibodies—particularly Fc-mediated effector functions—remains incompletely defined. We evaluated the immunogenicity and functional antibody responses induced by Ad26- and MVA-based HIV-1 mosaic vaccines in SHIV-infected rhesus macaques and ART-suppressed people with HIV. In nonhuman primates, vaccination significantly increased Env-specific antibody titers, Fcγ receptor binding, and Fc-dependent functions, including cellular phagocytosis, complement deposition, and NK cell activation. Responses peaked following MVA boosting and, although they declined over time, remained elevated compared with unvaccinated controls. Humoral responses did not predict viral rebound during analytic treatment interruption but correlated inversely with post-ART viral setpoints, suggesting a role in viral control. In a parallel human study, therapeutic vaccination similarly elicited functional antibody responses, with the strongest effects observed following Ad26 mosaic vaccination combined with a gp140 protein boost, whereas Ad26 and MVA alone induced more modest responses. Ad26-based HIV vaccines, especially with protein boosting, elicit robust, multifunctional antibody responses; although human virologic outcomes remain untested, these findings support exploring Fc-mediated humoral immunity for viral control and cure strategies.

## Introduction

Elimination of the latent HIV-1 reservoir via immunotherapeutic interventions is being evaluated as an HIV-1 cure strategy^1^. This includes the use of therapeutic immunization to enhance the anti-HIV immune response with the goal of inducing a state of immune-mediated viral control thereby rendering continuous antiretroviral therapy obsolete. To date, therapeutic vaccination approaches have primarily aimed at the induction of broad and potent anti-viral T-cell responses, specifically cytotoxic CD8^+^ T-cells, with the goal to kill cells harboring the HIV reservoir^2^. The elicitation of HIV-specific antibodies by therapeutic vaccine candidates, however, has received less attention, and reported data have been primarily around Env-specific binding antibody titers (reviewed in^2^). Potentially relevant for HIV viral eradication efforts, antibodies can mediate a multitude of functions via their fragment crystallizable (Fc) region, including recruitment and activation of innate effector cells via the engagement of cell-surface expressed Fc gamma receptors (Fcγ-R) that have the ability to deplete infected target cells^3–7^. These Fc effector functions have been shown to be important in protection against multiple pathogens, including HIV-1, influenza, SARS-CoV-2, Ebola, Dengue, HPV etc.^8–18^. Specifically, we and others have shown that antibodies able to mediate innate effector activities via binding to Fcγ-Rs have been linked to delayed viral rebound in previous analytical treatment interruption (ATI) studies^19,20^ suggesting that therapeutic enhancement of similar responses could be beneficial. If highly Fc-functional antibodies are induced by therapeutic vaccine approaches and if such responses contribute to viral control remains to be determined.

Adenovirus serotype 26 (Ad26) and Modified Vaccinia Ankara (MVA) vectored HIV vaccines, expressing bioinformatically optimized HIV-1 ‘mosaic’ Pol, Env, and Gag antigens, have been originally developed for the prevention of HIV infection^21^. In addition to the induction of robust T-cell responses in ref. ^22^, previous studies have demonstrated that these vaccine regimens were able to induce functional antibodies in naïve rhesus macaques, and these antibodies, after adoptive transfer, were able to protect naïve animals against Simian-Human Immunodeficiency Virus (SHIV) challenge^23^. Ad26 and MVA vaccines, in the following called Ad26/MVA, administered to SIV-infected rhesus macaques during antiretroviral therapy (ART) suppression resulted in reduced viral load set points when combined with a TLR agonist^24^ following ART interruption (ATI), highlighting their potential in enhancing immune-mediated viral control. These vaccines were also safe and well tolerated in individuals who initiated ART during acute HIV-1 infection (Fiebig stages I‒IV) in the RV405 study, resulting in increased HIV-specific immune responses compared to placebo treatment^25^.

We recently had evaluated the therapeutic use of the Ad26/MVA mosaic vaccines in 2 studies: Ad26/MVA mosaic vaccines and the broadly neutralizing HIV-1 antibody PGT121 in addition to the TLR7 agonist Vesatolimod were administered to SHIV-_SF162P3_ infected ART suppressed macaques. Following ATI, 70% of the animals from the Ad26/MVA plus PGT121 achieved viral control with undetectable SHIV viral loads, while in the PGT121 only, Ad26/MVA only, and sham groups viral control was reached in 41.6%, 33.3%, and 0% of animals, respectively^26^. The second study, HTX1002, did not include an ATI but focused on immunogenicity of 2 therapeutic vaccine regimens in people living with HIV-1 (PLWH) that were virologically suppressed on ART: either two doses of Ad26.Mos4.HIV and two doses of MVA-Mosaic were administered, resembling the vaccination regimen in the NHP study, or participants received two doses of Ad26.Mos4.HIV and two doses of Ad26.Mos4.HIV plus adjuvanted Clade C (C97ZA012) Env gp140 and Mosaic Env gp140 vaccines^27^. In this study, both vaccine regimens were immunogenic; however, the addition of the protein boost resulted in augmented binding antibody titers.

To further explore the potential of these therapeutic vaccinations to elicit functional antibodies responses in both Simian and Human Immunodeficiency Virus infection and to determine the role of such responses in viral control post ART interruption in the NHP study, we applied a System’s Serology approach to perform a deep characterization of longitudinal humoral immune profiles in the 49 animals for the NHP study and in the 25 participants of the HTX1002 study throughout the course of treatment interventions. Specifically, we profiled antigen-specific antibody isotype and subclass titers, Fcγ-R binding, and Fc effector functions (including antibody-dependent complement deposition, neutrophil and monocyte phagocytosis, and NK cell activation) and correlated the antibody signatures with clinical outcomes.

## Results

### Non-human primate

**Fig. 1:**
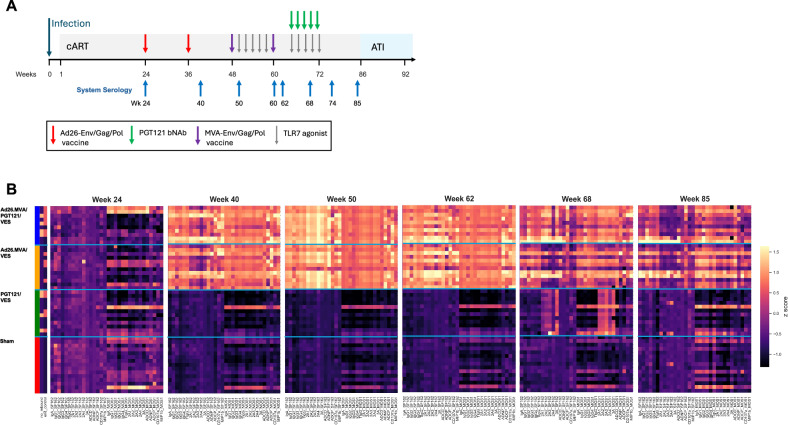
Comprehensive systems serology data of the tested non-human primates. **A** Non-human primate study design. Time points at which system’s serology analysis was performed are highlighted by blue arrows. **B** Heatmap of Z-scored data of the antibodiome data set (antibody titer, Fcγ-R binding, ADCD, ADNP, ADCP, ADNKA) for SF162 and Mos-1 antigens in rhesus macaque at different timepoints during the study.

For the comprehensive and longitudinal analysis of immunotherapy-elicited humoral response profiles throughout the course of active and passive immunization in the NHP study, we selected plasma samples from 8 time points during continuous ART: at week 24 following SHIV infection but prior to Ad26 vaccination, four weeks after the second Ad26 dose (week 40), two weeks after initiation of TLR7 stimulation and first MVA vaccine (week 50), at the day of the second MVA vaccine (week 60), during PGT121 administration (week 62 and week 68), two weeks after completion of PGT121 and TLR7 stimulation treatment (week 74) and prior to ART cessation (week 85) (Fig. 1a).

#### Ad26/MVA vaccination elicits robust antibody titers, with increased Fcγ-R binding and Fc-mediated functional responses

**Fig. 2:**
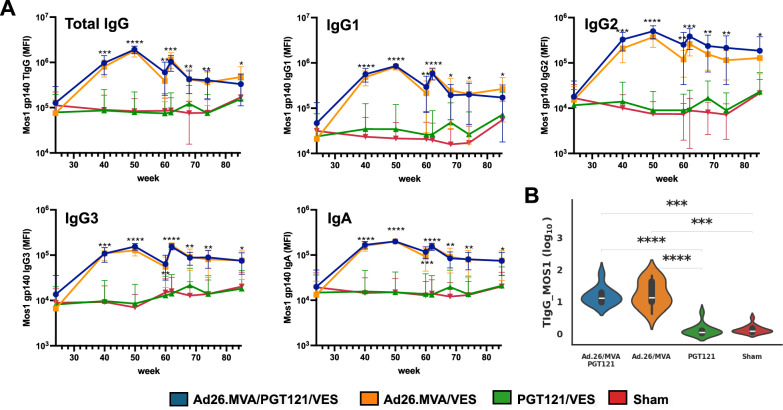
Kinetics of Mos-1 gp140-specific antibody levels following therapeutic vaccination in the NHP study. **A** Levels of Mos-1 gp140-specific total IgG, IgG1-3 and IgA1 levels throughout the study. The different study arms are color-coded. MFI, mean fluorescence intensity. Asterisks indicate statistically significant difference between the groups using one way ANOVA test between the different groups for each timepoint with Benjamini-Hochberg correction (*, *P* ≤ 0.05; **, *P* ≤ 5E-5; ***, *P* ≤ 5E-10; ****, *P* ≤ 5E-15). **B** Area under the curve (week 24 to week 85) of Mos-1 gp140-specific total IgG1 levels comparing animals that had received Ad26/MVA + PGT121, Ad26/MVA, PGT121 or placebo. Asterisks indicate statistically significant differences between the groups using Dunn’s test (*, *P* ≤ 0.01; **, *P* ≤ 0.001; ***, *P* ≤ 0.0001; ****, *P* ≤ 0.00001).

Baseline antibody profiles at week 24, prior to immunotherapeutic interventions, were comparable between all animal suggesting homogenous induction of anti-SHIV humoral immunity in response to infection (Fig. 1b). Following Ad26/MVA vaccination, we observed a statistically significant increase in IgG1-3 and IgA gp140 Mos1-, and gp120 SF162-specific responses across all vaccinated macaques (Fig. 2a, Supplementary Fig. 1a) which remained significantly higher than titers in the unvaccinated or PGT121 only treated animals (Supplementary Fig. 2). Titers for all measured isotypes and subclasses in vaccinated animals peaked after the administration of the first MVA vaccine dose but although they displayed a continuous decrease over time, responses remained substantially higher than in the Sham and PGT121 only groups throughout the treatment course. Calculating the Area-under-the-curve (AUC) for antibody titers, including measurements from week 24 to week 85, the pooled AUC were significantly higher between the vaccinated animal groups and the sham or PGT121 only groups, with no significant difference between the latter two (Fig. 2b, Supplementary Fig. 1b). When comparing animals that had only received the Ad26/MVA vaccines with animals that had in addition received PGT121, we did not notice any differences, specifically there was no enhancing effect of PGT121 on the vaccine boosted antibody response (Fig. 2b, Supplementary Fig. 1b). For the purpose of this study, our assays used NHP antibody specific detection reagents as we were interested in the macaque autologous immune response and it should be noted that when we measured week 68 titers (the study period during which PGT121 was administered) with human antibody targeting reagents, we saw a significant increase in total SF162 binding IgG that rapidly declined as PGT121 washed out (data not shown).

**Fig. 3:**
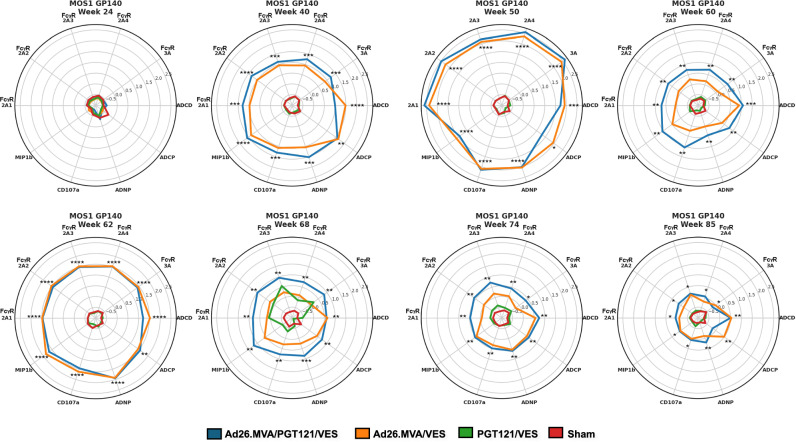
Kinetics of Fcγ-R binding and functional antibodies. Radar plot representation of the Z-scored Fcγ-R binding and antibody functions for the tested antigen Mos-1 gp140 at multiple timepoints throughout the study. The different study arms are color-coded. MFI, mean fluorescence intensity. Asterisks indicate statistically significant difference between the groups using one way ANOVA test between the different groups for each timepoint with Benjamini-Hochberg correction (*, *P* ≤ 0.05; **, *P* ≤ 5E-5; ***, *P* ≤ 5E-10; ****, *P* ≤ 5E-15).

A similar trend as for antibody titers was observed for Fcγ-R binding activity, where vaccinated animals showed an increase in Fcγ-R 2A1-4, and 3 A binding post-vaccination (Fig. 3, Supplementary Figs. 3 and 4). Receptor binding dynamics were again similar between the groups that received vaccine and PGT121 or vaccine alone (R^2^ = 0.89–0.98, Supplementary Table 1). While no expansion of Fcγ-R binding antibodies was seen in the sham group, modest increase was seen in the PGT121 only group at week 68 during active bNAb dosing. PGT121, a human IgG1 binds to human Fcγ-Rs, however, affinity human antibody binding to macaque Fcγ-Rs can be quite variable^28^ possibly explaining the only modest expansion of Fcγ-R binding activity despite robust plasma levels of PGT121. Overall, increase in Fcγ-R binding magnitude appeared to be more transient, diminishing towards the end of follow up despite persistently elevated antibody titers (Fig. 3, Supplementary Figs. 3 and 4). Consistent with the robust expansion of antibody responses with Fcγ-R binding activity, we observed significant induction of antigen specific antibody dependent complement deposition (ADCD), cellular phagocytosis (ADCP), neutrophil phagocytosis (ADNP) and NK cell activation (ADNKA), as determined by CD107a and/or MIP-1β expression in the vaccinated animals (Fig. 3, Supplementary Figs. 3 and 4). Functional response kinetics followed overall titer levels reaching peaks at the time of MVA doses but decreased rapidly afterwards although and with the exception of ADCP, they remained significantly higher in the vaccinated animals compared to the sham or PGT121 only group on the day of ART cessation (Dunn’s test in vaccinated vs unvaccinated groups: ADCD *p* < 0.001; ADNP *p* < 0.001; CD107a *p* < 0.01; MIP-1β *p* < 0.01). During the PGT121 administration periods we did only observe minor increases in serum-mediated ADCD and CD107a expression on NK-cells in the PGT121 only treated animals, suggesting suboptimal Fc-mediated PGT121 effects consistent with the modest Fcγ-R binding activity observed in this group.

**Fig. 4:**
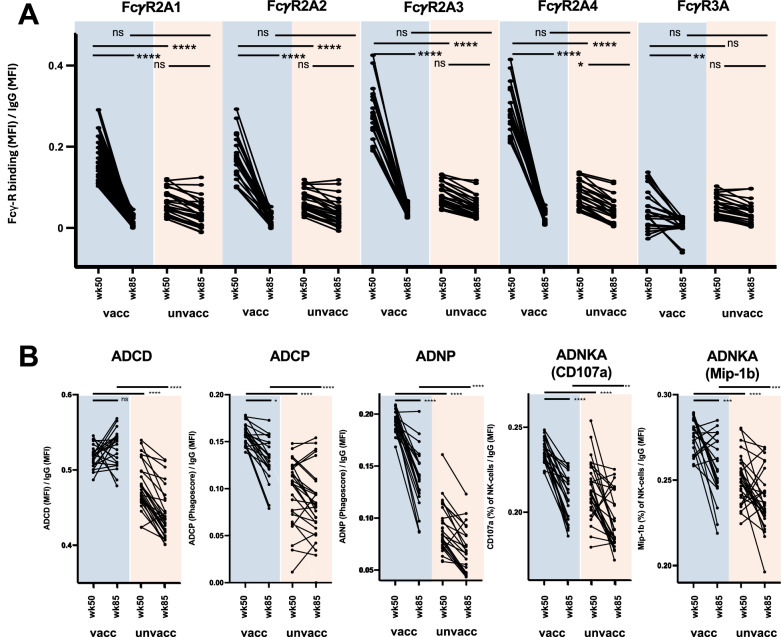
Changes in antibody quality between week 50 and 85. Serum Fcγ-R binding and antibody functions at week 50 and week 85 in animals that had received the therapeutic vaccine versus animals that only received PGT121 or placebo. Serum Fcγ-R binding (**A**) and functions (**B**) were corrected for the animals Mos-1 gp140-specific total IgG titer (as determined by Luminex) MFI, mean fluorescence intensity. Asterisks indicate statistically significant differences between the groups in the univariate test after correction for multiple comparison (*, *P* ≤ 0.05; **, *P* ≤ 0.01; ***, *P* ≤ 0.001; ****, *P* ≤ 0.0001).

To further explore the apparent disconnect between persistent antibody titers over time and decreasing Fcγ-R binding and functional activity, we corrected these features by total IgG titers according to antigen^29,30^, allowing us to assess these functionalities qualitatively on a per antibody level. We first confirmed that across all groups and prior to vaccination (week 24) antibodies were qualitatively similar with regards to Fcγ-R binding and functional activity (data not shown). Subsequently, during peak immunogenicity at week 50, we observed significantly elevated binding to Fcγ-R2A1-4 but not Fcγ-R3A (Fig. 4a) as well as increased ADCD, ADCP, ADNP and ADNKA activity per antibody in the vaccinated animals (Fig. 4b). However, these high-quality antibodies declined over the course of the study and were significantly reduced by week 85, with the exception of ADCD levels, which persisted and, in some cases, continued to increase.

Despite the overall decline of functional antibody levels over time, ADCD, ADCP, ADNP and ADNKA activity remained significantly higher at week 85 compared to responses observed in the unvaccinated animals (Fig. 4). These data suggest that, although Ad26/MVA vaccine administration during ART treatment elicited a broad humoral response—characterized by increased antibody titers and functional activity—the quality of the induced antibodies was relatively short-lived.

#### Humoral immune features correlate with post-ART viral load setpoints, but not with viral rebound

As the Ad26/MVA vaccination and PGT121 treatment had resulted in substantial rates of post-ART virologic control, most considerably when combined^26^, we set forth to investigate the relationship between humoral vaccine response signatures and post-ART virological outcomes. Specifically, we examined whether antibody features correlated with viral control, time to viral rebound, and post-ART RNA viral load setpoints as previously reported^26^ (Supplementary Fig. S5).

**Fig. 5:**
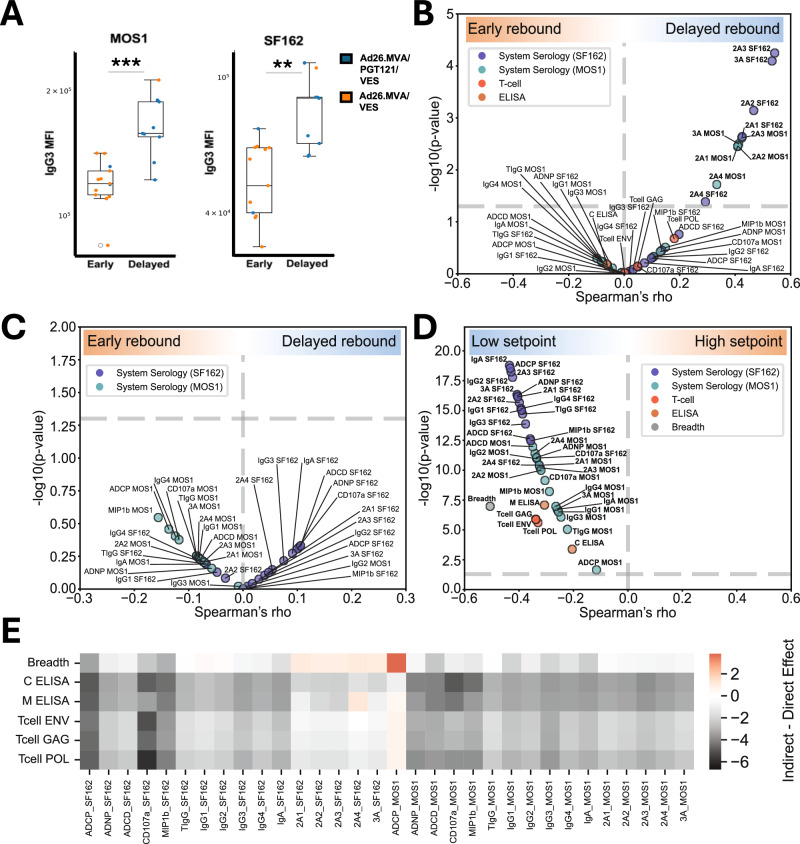
Correlations of antibody features with virological outcomes. **A** SF162 and Mos1-gp140-specific IgG3 levels differences at week 50 between vaccinated animals with early (within 14 days from ARTI) or delayed viral rebound. Means with interquartile ranges are shown in boxplots. Dots are color coded to reflect different groups. Asterisks indicate statistically significant differences between the groups using Mann–Whitney’s U test (*, *P* ≤ 0.01; **, *P* ≤ 0.001; ***, *P* ≤ 0.0001) MFI, mean fluorescence intensity (**B**) Spearman’s rho correlation of Fcγ−R binding activity (measured at week 68, concurrent with PGT121 administration) and of T‑cell and ELISA responses (measured at week 74) with days to viral rebound. **C** Spearman’s rho correlation of AUC summations of antibody features with time to viral rebound. **D** Spearman’s rho correlation of all available time‑points for antibody effector functions (System Serology), clade C and group M mosaic Env binding antibody levels, and T‑cell responses (breadth and Gag/Pol/Env magnitude) with viral‑load setpoint. **E** Heatmap of the difference between indirect (mediated) and direct effects of the different immune features, where rows represent potential mediators (T‑cell magnitude and breadth, bidning antibody titers by ELISA) and columns represent biophysical and Fc‑effector features; positive values (red) indicate larger indirect effects, negative values (black) indicate larger direct effects.

We first focussed on the antibody features at 2 timepoints: at peak immunogenicity (week 50) and directly prior to the ATI (week 85) (Supplementary Fig. 6a and b). Isolated antibody signatures at these time points did not predict viral rebound status by univariate Mann–Whitney analysis or by multidimensional Partial Least Squares Discriminant Analysis (PLS-DA) (Supplementary Fig. 6c). This was not surprising as all animals in the Ad26/MVA only or Sham only groups had rebounded while animals without rebound only appeared in the groups that included PGT121 administration, strongly suggesting that the bNAb rather than the vaccines affected rebound status. We also did not see any significant differences in antibody profiles between rebounding and non-rebounding animals in the group that had received both vaccines and bNAb, but numbers were small overall (6 versus 4; data not shown). However, in the 22 vaccinated animals at peak immunogenicity and prior to PGT121 administration (week 50), IgG3 levels were significantly higher (Mann–Whitney, IgG3 MOS1 GP140 *p* = 0.0003, IgG3 SF162 GP120 *p* = 0.001, (Fig. 5a) in animals who did not rebound after ART cessation, suggesting a possible role for IgG3 in predicting vaccine mediated viral control.

When assessing time-to-rebound, delays beyond 14 days post-ART interruption occurred only in groups receiving PGT121, not vaccines alone. Indeed, Fcγ−R binding activity at week 68, when PGT121 was administered was directly correlated with days to viral rebound (Fig. 5b). As this effect had waned by week 85, prior to ATI, our data suggests that the bNAb potentially mediated an anti-reservoir effect during the dosing phase subsequentially resulting in prolonged viral control.

To capture the cumulative impact of humoral responses, we next calculated AUC values for antibody features across all time points and correlated these with virological outcomes. Excluding animals that failed to rebound, we calculated Spearman’s correlations with SHIV RNA viral load setpoints, that had previously been defined in the original report of the NHP study^26^. AUC summations of vaccine induced humoral responses did not correlate with time to viral rebound (Fig. 5c), as this parameter appears to also be predominantly influenced by PGT121 administration, although neutralizing activity was not detectable anymore at the time of ATI as previously reported^26^.

In contrast, multiple antibody features were inversely correlated with viral load setpoint (Spearman’s rho = −0.62 to −0.4, *p* < 0.00005 to <0.05), including antibody titers, Fcγ−R-binding activity and several antibody-mediated functions (Fig. 5d). Amongst other measured features, ADCD and NK-cell activation were also associated with lower viral load set points (ADCD: *r* = −0.38, *p* < 0.01, CD107a: *r* = −0.41, *p* < 0.01, Mip-1β: *r* = −0.36, *p* = 0.01). As Walker-Sperling et al. had previously described that the breadth and magnitude of T-cell responses to Env, Pol, and Gag epitopes as measured by ELISPOT as well as binding antibody titers against both the HIV-1 Clade C and group M mosaic Env measured by ELISA correlated with setpoint viral loads following ART interruption^26^ we included these variables, where available, into our analysis. We confirmed the correlation of the T-cell and binding antibody with viral load but also noticed that most antibody Fc-signatures exhibited higher correlation coefficients, especially those measured against SF162. A mediation analysis aimed at disentangling the effects of T-cell and antibody Fc signatures further revealed that T-cell breadth, magnitude and binding antibody titers, generally did not mediate the effect of antibody Fc-signatures on viral load (Fig. 5e).

Lastly, we evaluated the performance of multiple Ordinary Least Squares (OLS) regression models incorporating different feature sets. Models based solely on Fc-functional antibody signatures derived from System Serology achieved among the highest ${R}^{2}$ values, second only to the model integrating System Serology, ELISA, and T-cell breadth (Supplementary Fig. 7a). In contrast, models including only T-cell or ELISA data performed substantially worse. Despite its strong explanatory power, the System Serology–only model exhibited a relatively high Akaike Information Criterion (AIC), indicative of overfitting compared with other models (Supplementary Fig. 7b, c), likely reflecting strong intercorrelations among System Serology features. Notably, inclusion of T-cell response magnitude or breadth, or ELISA measurements, reduced the AIC by more than 50%, indicating that these datasets contribute independent and complementary information to viral load prediction.

These data suggest that while vaccination alone may not be sufficient to predict viral rebound, vaccine enhanced humoral responses might contribute to better long-term control of the virus.

### People living with HIV

**Fig. 6:**
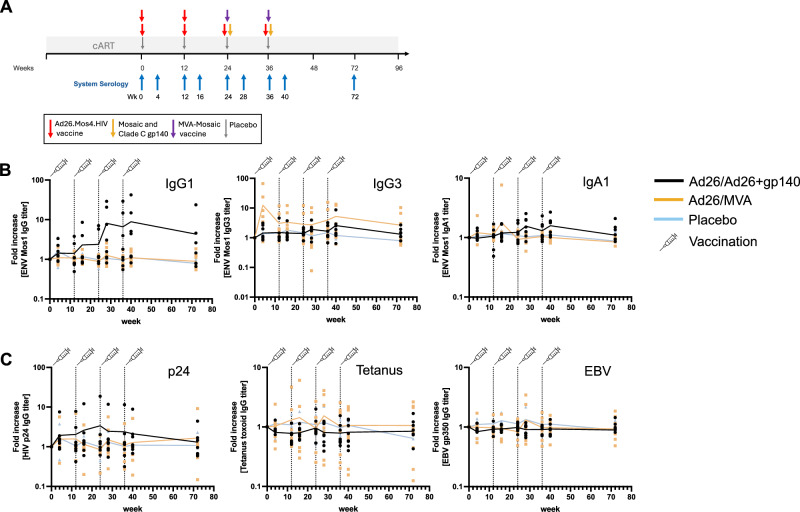
Kinetics of Mos-1 Env-specific antibody levels following therapeutic vaccination in the HTX1002 study. **A** HTX1002 study design. Time points at which system’s serology analysis was performed are highlighted by blue arrows. **B** Fold change (to baseline – week 0) Mos-1 Env-specific IgG1, IgG3 and IgA1 levels throughout the study. **C** Fold change (to baseline – week 0) of total IgG levels specific for HIV-1 p24, tetanus toxoid or EBV, respectively. The different study arms are color-coded. The vertical dotted lines represent the timepoints of vaccine/placebo administration.

Given the significant enhancement of Fcγ-R binding and Fc effector function mediating antibodies following therapeutic vaccination in SHIV-infected macaques we were curious if similar antibody expansion would be induced in PLWH. We therefore next applied our System Serology approach to the 25 participants of the HTX1002 clinical trial^27^. We selected 9 timepoints throughout the course of the study, specifically, week 0 (prior to vaccination), week 4, week 12, week 16, week 24, week 28, week 36, week 40, and week 72 (Fig. 6a).

#### Therapeutic vaccination elevates functional antibodies in humans

Given the substantial heterogeneity in baseline antibody responses within this human cohort—likely due to variability in duration of HIV infection prior to ART initiation and diversity in transmitted founder viruses—compared to the NHP study, we assessed vaccine effects by calculating the fold change in antibody features from the pre-vaccination baseline (Fig. 6b, Supplementary Fig. 8). The Ad26/MVA regimen had minimal impact on IgG1 responses to Mos1 Env, with a transient increase in IgG3 observed. In contrast, the Ad26/Ad26+gp140 regimen induced a substantial increase in IgG1 responses with no significant IgG3 induction. Neither vaccine affected IgA1 responses, and no changes were observed in the placebo group. Furthermore, no alterations in p24-specific antibodies or non-HIV antibody responses (assessed via tetanus toxoid and EBV-specific titers) were detected (Fig. 6c).

**Fig. 7:**
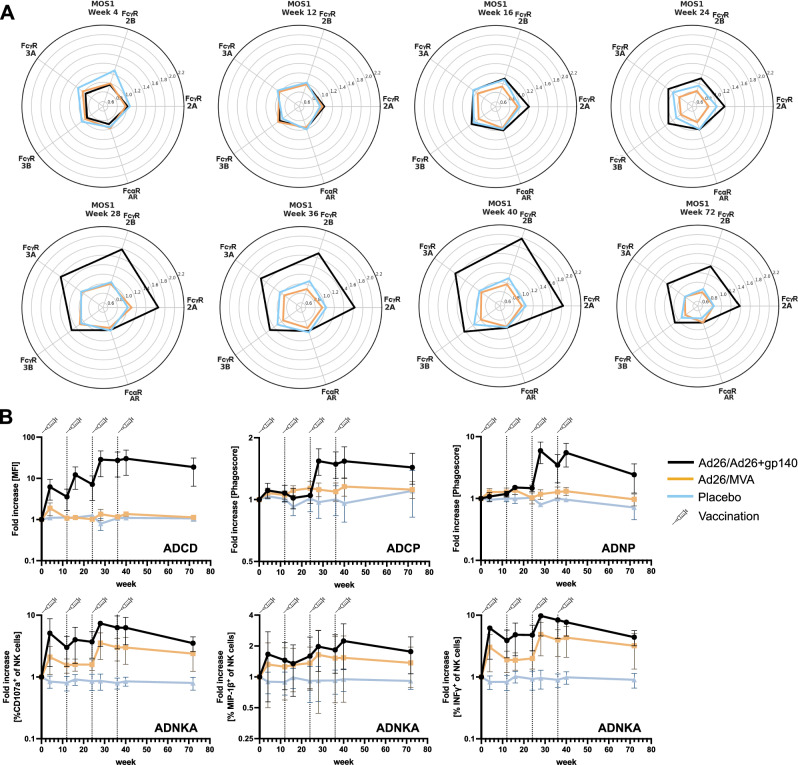
Kinetics of Fcγ-R binding and functional antibody response following therapeutic vaccination in the HTX1002 study. **A** Radar plot representation of the Z-scored Fcγ-R (2 A, 2B, 3 A, 3B) and Fcα-R (AR) binding activity of Mos-1 Env-specific antibody responses throughout the study. **B** Fold change (to baseline – week 0) functional response levels for Mos-1 Env-specific ADCD, ADCP, ADNP and ADNKA (as determined by CD107a expression or Mip-1β or IFNγ secretion) throughout the study. The different study arms are color-coded. The vertical dotted lines represent the timepoints of vaccine/placebo administration.

Fcγ-R binding activity was not altered by either the Ad26 vaccine or the MVA-vaccine boost. However, following Ad26+gp140 administration, a significant increase in binding activity to Fcγ-R2A, Fcγ-R2B, and Fcγ-R3A was observed. This increase peaked shortly after administration and gradually declined towards week 72, although it remained detectable at that time (Fig. 7a). In contrast, Fcα receptor (Fcα-R) binding was unaffected by any of the vaccine regimens as expected from the stable IgA titers. Enhancements in ADCD, ADCP, and ADNP were primarily observed in recipients of the Ad26/Ad26+gp140 vaccine regimen, as previously reported in part for ADCP^27^ (Fig. 7b, Supplementary Fig. 8). Both the Ad26/MVA and Ad26/Ad26+gp140 regimens increased NK-cell activating antibodies, with the latter regimen demonstrating a more pronounced effect.

These results suggest that Ad26-based vaccines, particularly when combined with a gp140 protein boost, enhance functional antibody responses. Since the design of the HTX1002 study did not include an ATI, the potential virological impact of these enhanced antibody responses remains undetermined.

## Discussion

The results of this study provide valuable insights into the immunological effects of therapeutic vaccination, particularly with Ad26/MVA mosaic vaccines, and their potential role in enhancing immune-mediated viral control. Previous studies have shown that therapeutic vaccination, often focused on T-cell responses, is a promising strategy to enhance anti-HIV immune responses with the goal to ultimately eliminate the latent HIV reservoir^1,2^. However, much less attention has been given to the role of humoral immune responses, including antibodies, in post-ART viral control, despite their ability to mediate important Fc-dependent effector functions which are likely critical for targeting infected cells^3–7^.

In the NHP model, the Ad26/MVA vaccination significantly boosted IgG and IgA antibody titers, and more importantly, it enhanced Fcγ-R binding and Fc-mediated immune responses. These humoral responses peaked shortly after the second MVA vaccine dose and were associated with higher functional antibody activities, including ADCD, ADCP, and NK-cell activation. Interestingly, while antibody titers remained elevated throughout the study, Fcγ-R binding activity and functional responses decreased over time, indicating that the quality of the induced immune response was transient. However, these responses were still superior to those observed in the sham or PGT121-only groups, suggesting that the Ad26/MVA vaccine regimen was effective at inducing functional humoral responses.

Previous NHP studies testing Ad26/MVA vaccine regimens for SIV/SHIV prevention of infection identified antibody functions including ADCP, NK cell activation and binding to certain Fcγ-Rs as correlates of protection^23^. The association between humoral immune features and post-ART virological outcomes was therefore another key aspect of our study. Notably, although vaccine-induced antibody signatures, including Fcγ-R binding activity, did not predict viral rebound status or time to viral rebound directly, specific antibody features were inversely correlated with viral load set points. In particular, IgG3 levels following vaccination were significantly higher in animals that did not experience viral rebound, suggesting a potential contribution of IgG3 to viral control. Together, these findings support the concept that while vaccine-induced humoral immunity alone may be insufficient to prevent viral rebound, enhanced functional antibody responses—especially those capable of engaging Fcγ receptors—may contribute to better viral control and delayed rebound after ART cessation.

Consistent with this framework, both measures of NK cell activation (CD107a and MIP1β) were significantly elevated in the vaccine group, whereas FcγR3A binding normalized to total IgG was not. This pattern suggests that increased NK cell activity may reflect the overall abundance of FcγR3A-binding antibodies, which scales with total IgG levels, as indicated by the significant increase observed prior to normalization. Importantly, the persistence of elevated NK activation markers after IgG correction implies that FcγR3A-mediated effects may not be strictly linear and that additional downstream or amplification mechanisms may enhance NK cell activation. Further investigation will be required to elucidate these pathways.

The role of PGT121 appeared to be important in modulating post-ART viral outcomes. Animals receiving both Ad26/MVA vaccines and PGT121 exhibited the longest delay in viral rebound, suggesting an additive or complementary effect of vaccine-elicited immune responses and antibody administration on post-treatment viral dynamics. Fcγ-R binding activity, particularly during the period of PGT121 administration, correlated with time to viral rebound, supporting a role for Fc-mediated antibody functions in influencing viral recrudescence. Although exogenously administered neutralizing antibody was not detectable at the time of ATI^26^, PGT121 may have influenced viral dynamics during the treatment phase in ways that impacted subsequent rebound kinetics. However, in the absence of direct measurements of the viral reservoir or assessment of vaccine coverage of reservoir sequences, these findings should be interpreted cautiously.

The clinical implications of these findings are further supported by data from the HTX1002 study in humans, where therapeutic vaccination induced modest increases in functional antibody responses, particularly following the Ad26/Ad26+gp140 regimen. Increases in Fcγ-R binding activity and antibody-dependent complement deposition (ADCD) were observed in participants; however, no direct virological impact could be assessed, as the study did not include an analytical treatment interruption (ATI). Additional evidence consistent with a potential contribution of functional antibody responses to virological outcomes following Ad26-based mosaic vaccination comes from the RV405 study, which reported higher ADCP responses in individuals who experienced a modest delay in viral rebound during ATI. Although the study population differed from ours in that ART was initiated during acute rather than chronic infection and vaccine coverage of rebound viruses was limited, these findings nonetheless suggest an association between Fc-mediated antibody function and delayed viral recrudescence, warranting further investigation across different clinical settings^31^.

Overall, these data highlight the importance of functional antibody responses, in addition to neutralizing antibodies, in controlling HIV-1 infection in the absence of ART. The combination of therapeutic vaccination and broadly neutralizing antibodies, particularly those that engage Fcγ-Rs and mediate cellular cytotoxicity, may offer a promising strategy for achieving sustained viral control, clearance of latently infected cells and moving closer to an HIV cure. However, further research is needed to better understand the timing, quality, and long-term durability of these responses, as well as their potential to complement existing ART strategies for HIV eradication.

## Methods

**NHP study:** plasma samples had been previously obtained from 49 adult rhesus macaques as part of an immunotherapy combination study^26^. The latter study had been approved by the appropriate Institutional Animal Care and Use Committee (IACUC) at Beth Israel Deaconess Medical Center and Bioqual. Animals had been infected intrarectally with a single 500 TCID50 dose of SHIV-SF162P3 challenge stock, and ART was initiated on day 9. Macaques had been divided into 4 treatment groups: sham, PGT121, Ad26/MVA vaccines (Ad26 vectors expressing SIV_smE543_ gag-pol, HIV-1 mosaic-1 env, and HIV-1 mosaic-2 env/ MVA expressing SIV_smE543_ gag-pol, HIV-1 mosaic-1 env-gag-pol, and HIV-1 mosaic-2 env-gag-pol) or PGT121 and Ad26/MVA vaccines. ART was continued until week 86 and was then interrupted. For our study, plasma samples from eight timepoint, specifically weeks 24, 40, 50, 60, 62, 68, 74, and 85 were included.

**Human study:** plasma samples had been previously obtained from 25 ART-suppressed PLWH as part of a double-blind, placebo-controlled phase 1 trial (IPCAVD013/HTX1002)^27^ in which participants were randomized to receive Ad26.Mos4.HIV/MVA-Mosaic (Ad26/MVA) or Ad26.Mos4.HIV/Ad26.Mos4.HIV plus adjuvanted gp140 protein (Ad26/Ad26+gp140) or placebo. This study did not include an ATI. The study had been performed in accordance with the Declaration of Helsinki, had been approved by the BIDMC Institutional Review Board, and the study was registered on ClinicalTrials.gov (NCT03307915). For our study, plasma samples from nine timepoint, specifically weeks 0, 4, 12, 16, 24, 28, 36, 40 and 72 were included.

### Antigens

Mos-1 gp140 was supplied by Janssen. SF162 gp120, gp350 (EBV) and p24 HXBc2 was obtained from Immune Technology and tetanus was obtained from MassBiologics.

### Antibody isotype, subclass and FcR binding

HIV-antigen specific antibody isotype and subclass titers, along with Fcγ-receptor binding were measured in plasma samples via Luminex multiplexing, as previously described^32^. In short, antigens were directly coupled to distinct Luminex bead regions (Luminex Corp, TX, USA) through Sulfo-NHS (Thermofisher) and EDC (Thermofisher) coupling. Plasma samples were diluted (Rhesus: 1:50 for isotype and subclass, 1:500 for FcγR2a-1, FcγR2a-2 FcγR2a-3 FcγR2a-4, 1:1000 for FcγR3a, human: 1:500 for IgG1 1:100 for remaining isotypes and subclasses and 1:1000 for FcγRs) and added to the coupled bead mix in 384 well plates (Greiner Bio-One, Germany) for a 2-h incubation at room temperature. excess antibodies were washed away, total IgG titers were detected with PE-conjugated antibody (Southern Bio #4700-09), IgG subclasses and IgA levels were measured using a secondary anti-rhesus antibody (NIH Nonhuman Primate Reagent Resources) and tertiary PE-conjugated antibody (ThermoFisher #31861). For FcγR binding, recombinant and site-specific (AVI-tag) biotinylated FCγR (Duke University Protein Core, USA) were coupled with PE-Streptavidin (Agilent Technologies, CA, USA) and used as a secondary probe. Following a 1-hour incubation and wash, immune complex median florescence intensity (MFI) per antigen was determined using an IQue analyzer (Intellicyt).

#### ADCD

Antibody-dependent complement deposition was measured as described previously^4^. In short, HIV biotinylated antigens were coupled with Neutravidin microspheres (Thermo Fisher F8775) and then incubated with NHP plasma samples (diluted 1:50) or human plasma samples (diluted 1:10) at 37 °C for 2 h. After incubation, non-specific antibodies were washed and guinea pig complement in GVB + + buffer (Boston BioProducts, MA, USA) was added for 20 min incubation at 37 °C. 15 mM EDTA-PBS Invitrogen #15575020) was used to stop complement reaction, and bead attached C3 was stained with anti-guinea pig C3-FITC antibody (MP Biomedicals, CA, USA) and analyzed by flow cytometry (NHP: Stratedigm 1300EXi., Human: Intellicyt iQue)

#### ADCP

Antibody-dependent Phagocytosis by THP-1 cell line human monocytes (ATCC #TIB-202) was measured as described previously^3^. In short, yellow-green fluorescent neutravidin microspheres (Thermo Fisher F8776) were coupled with HIV biotinylated antigens and incubated with NHP or human plasma samples (diluted 1:100) for two hours. Following incubation THP-1 were added (0.25 × 10^5^ cells/well) and incubated overnight at 37 °C. After incubation cells were fixed with 4% paraformaldehyde (Santa Cruz Biotechnology #SC-281692) and analyzed by flow cytometry (NHP: Stratedigm 1300EXi., Human: Intellicyt iQue). Phagoscore was calculated by multiplying percentage of microsphere-positive cells by MFI of microsphere-positive cells and dividing by 100,000.

#### ADNP

Antibody-dependent primary human neutrophil phagocytosis was determined via a microsphere-based phagocytic assay, as described previously^6^. In short, HIV biotinylated antigens were coupled with Neutravidin microspheres (Thermo Fisher F8776) and then incubated with NHP or human plasma samples (diluted 1:50) at 37 °C for 2 h. ACK lysis buffer (Thermofisher A1049201) was used to derive primary human neutrophils from healthy donor whole blood samples. Neutrophils were then added to the antigen-bead immune complexes (5 × 10^4^ cells/well) and incubated for 1 h at 37 °C. Neutrophils were then stained with anti-Cd66b Pac blue antibody (BioLegend 305,112) and fixed with 4% paraformaldehyde (Santa Cruz Biotechnology #SC-281692). Analysis was performed by flow cytometry (NHP: Stratedigm 1300EXi., Human: Intellicyt iQue). Phagoscore was calculated by multiplying percentage of microsphere- CD66b positive cells by MFI of microsphere-positive cells and dividing by 100,000.

#### ADNKA

Antibody-dependent NK-cell activation was measured as previously described^33^. Flat-bottom 96-well ELISA plates (Thermo Fisher #439454) were coated with HIV antigen and incubated at 37 °C for 2 h and then blocked with 5% BSA (Sigma-Aldrich) in PBS, followed by an o/n incubation at 4 °C. NK cells were isolated from human peripheral blood leukopaks (Stemcell Technologies) using EasySep™ Human NK Cell Isolation Kit (Stemcell Technologies), stimulated with 1 ng/ml rhIL-15 (STEMCELL Technologies) and left to incubate o/n at 37 °C. After incubation, plates were washed 3 times with PBS and 50 µL of diluted (1:50) NHP or human plasma samples were added per well, followed by a 2-h incubation at 37 °C. Plates were washed and 5 × 10^4^ NK cells were added per well and incubated for 5 h at 37 °C with an inhibition cocktail composed of CD107a-PeCy5 (BD Biosciences, #555802), Brefeldin A (Sigma -Aldrich) and Golgistop^tm^ protein transport inhibitor (BD Biosciences). Cells were then transferred to V-bottom 96 well plates and surface stained for CD56 (BD, 1:200, clone: B159), CD16 (BD, 1:200, clone: 3G8), and CD3 (BD, 1:800, UCHT1). FIX & PERM Cell Permeabilization Kit (Thermo Fisher) was used for cell fixation and permeabilization. Cells were then intracellularly stained for MIP-1β (BD, 1:50, clone: D21-1351) and IFNγ (BD, 1:17, clone: B27). Marker frequencies were measured by flow cytometry (NHP: Stratedigm 1300EXi., Human: Intellicyt iQue).

### Statistical analyses overview

Kruskal-Wallis, Dunn’s test for multiple comparisons, and Spearman correlation tests were performed using Python version 3.11.1 scikit_posthocs, scipy.stats packages. Mann–Whitney tests were done with R Studio software version 2023.09.1 + 494. *Multivariate analysis was done using the systemseRology R package (version 1.0;* https://github.com/LoosC/systems_seRology)***.

#### Multivariate analysis

Before building models, the data were centered and scaled. For each of SF162- and Mos-1-specific features, partial least square-discriminant analysis (PLS-DA) was performed to build a classification model. Model accuracy was calculated with 5-fold cross-validation, and significance was evaluated by permutation testing.

#### Mediation analysis

Before building models, the data were centered and scaled. For each of SF162- and Mos-1-specific features considered as an independent variable $X$, the direct and indirect effects mediated through T‑cell, breadth, or ELISA variables $M$ on outcome $Y$ were calculated via a series of linear regressions:

$$\begin{array}{c}Y=\,{a}_{0}+{a}_{1}X+{\epsilon }_{1}\\ M={b}_{0}+{b}_{1}X+\,{\epsilon }_{2}\\ Y={c}_{0}+\,{c}_{1}X+\,{c}_{2}M+{\epsilon }_{3}\end{array}$$

The indirect (mediated) effect is calculated as the product ${b}_{1}\times {c}_{2}$, and the direct effect is ${c}_{1}$. Statistical significance of the indirect effect was assessed using the Sobel test.

#### Multivariable linear regression

Before building models, the data were centered and scaled. Ordinary Least Squares (OLS) regression was implemented. Model performance was evaluated using adjusted R^2^ and Akaike Information Criterion (AIC).

## Supplementary information

Supplementary Information

## Data Availability

All data associated with this study are present in the paper, the Supplementary Materials or available under [doi.org/10.7910/DVN/MA5X4Y].
