# Supplementary material for: Functional antibody signatures following therapeutic immunization in Simian and Human immunodeficiency virus infection
Source: NPJ Vaccines. 2026 Jan 31;11:62. doi: 10.1038/s41541-026-01390-3 (PMC12963365; doi:10.1038/s41541-026-01390-3)
Supplement: Supplementary file 1 — Supplementary Information [file 41541_2026_1390_MOESM1_ESM.pdf]

Supplemental Materials

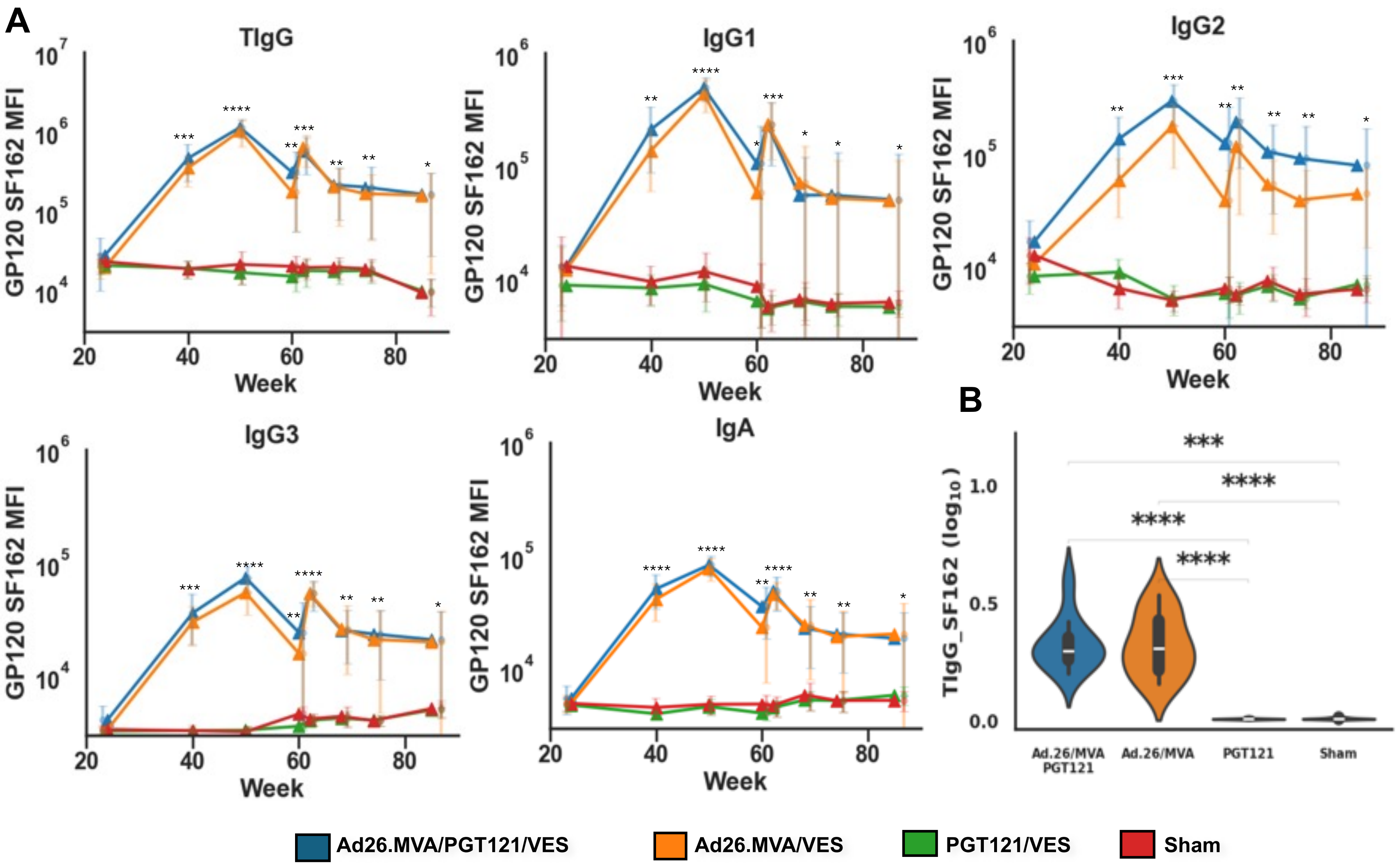

Supplementary Figure 1: Kinetics of SF162 gp120-specific antibody levels following therapeutic vaccination in the NHP study. (A) Levels of SF162 gp120-specific total IgG, IgG1-3 and IgA1 levels throughout the study. The different study arms are color-coded. MFI, mean fluorescence intensity. Asterisks indicate statistically significant difference between the groups using one way ANOVA test

between the different groups for each timepoint with Benjamini-Hochberg correction (*, $P \leq 0.05$; **, $P \leq 5E-5$; ***, $P \leq 5E-10$; ****, $P \leq 5E-15$). (B) Area under the curve (week 24 to week 85) of SF162 gp120-specific total IgG1 levels comparing animals that had received Ad26/MVA+PGT121, Ad26/MVA, PGT121 or placebo. Asterisks indicate statistically significant differences between the groups using Dunn's test (*, $P \leq 0.01$; **, $P \leq 0.001$; ***, $P \leq 0.0001$; ****, $P \leq 0.00001$).

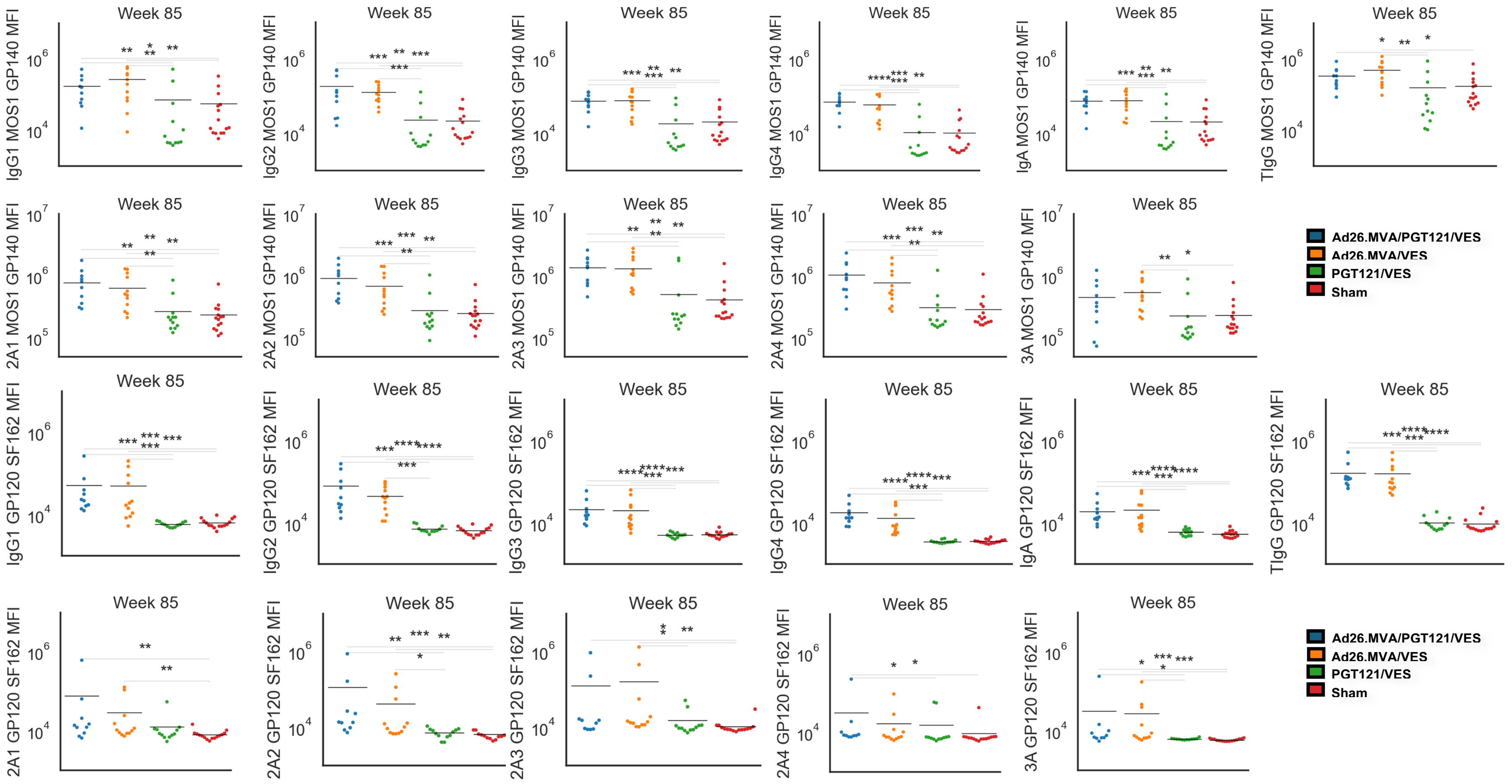

Week 85
IgG1 MOS1 GP140 MFI
IgG2 MOS1 GP140 MFI
IgG3 MOS1 GP140 MFI
IgG4 MOS1 GP140 MFI
IgA MOS1 GP140 MFI
TIgG MOS1 GP140 MFI
2A1 MOS1 GP140 MFI
2A2 MOS1 GP140 MFI
2A3 MOS1 GP140 MFI
2A4 MOS1 GP140 MFI
3A MOS1 GP140 MFI
IgG1 GP120 SF162 MFI
IgG2 GP120 SF162 MFI
IgG3 GP120 SF162 MFI
IgG4 GP120 SF162 MFI
IgA GP120 SF162 MFI
TIgG GP120 SF162 MFI
2A1 GP120 SF162 MFI
2A2 GP120 SF162 MFI
2A3 GP120 SF162 MFI
2A4 GP120 SF162 MFI
3A GP120 SF162 MFI
Ad26.MVA/PGT121/VES
Ad26.MVA/VES
PGT121/VES
Sham

Supplementary Figure 2: Total IgG, IgG1-3, IgA1 and Fcγ-R binding to Mos1-gp140 or SF162-gp120 at week 85 between animals receiving Ad26/MVA/PGT121/VES, Ad26/MVA/VES, PGT121/VES or placebo. Asterisks indicate statistically significant differences between the groups using Dunn's test (*, $P \leq 0.01$; **, $P \leq 0.001$; ***, $P \leq 0.0001$; ****, $P \leq 0.00001$).

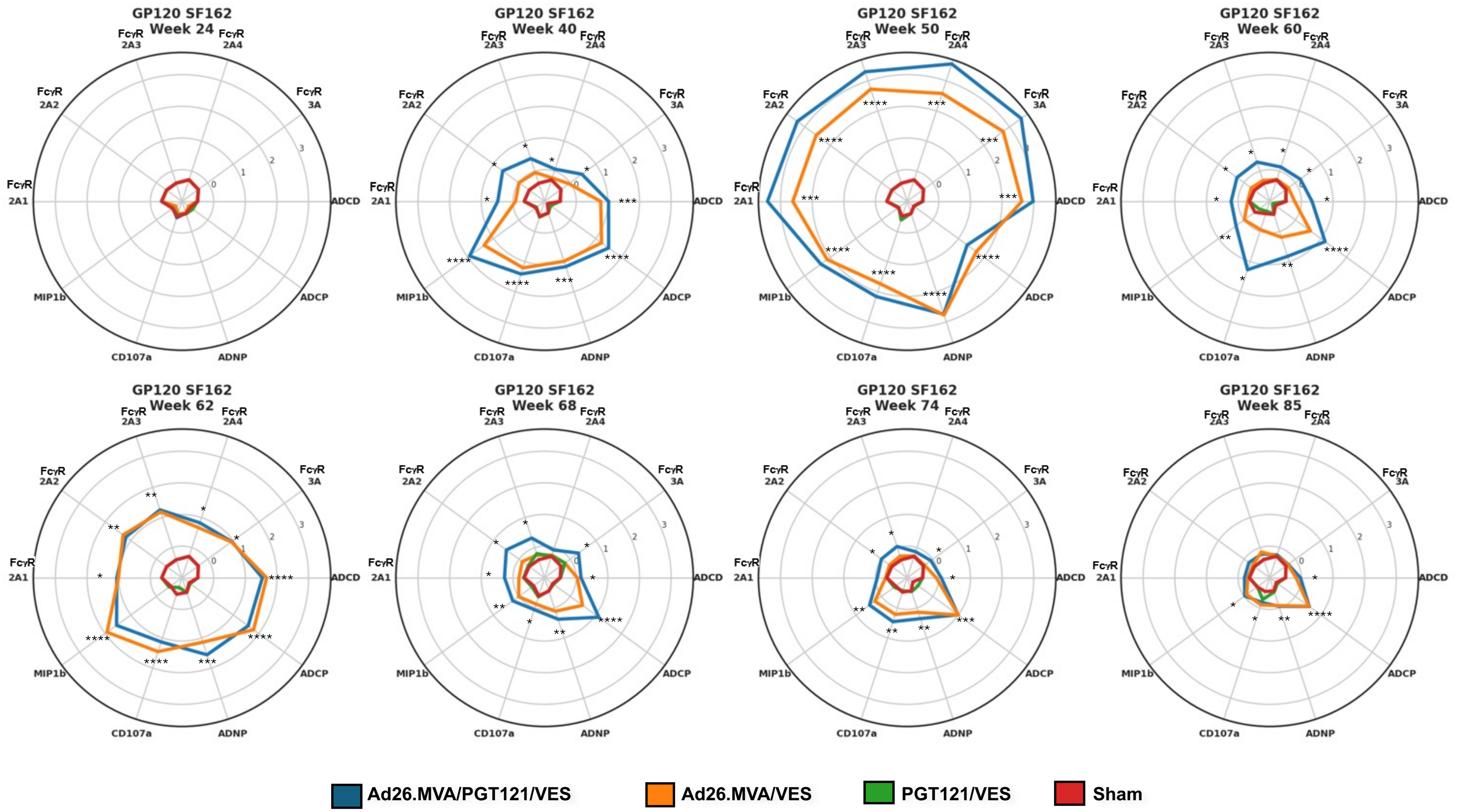

Supplementary Figure 3: Kinetics of Fcγ-R binding and functional antibodies. Radar plot representation of the Z-scored Fcγ-R binding and antibody functions for the tested antigen SF162 gp120 at multiple timepoints throughout the study. The different study arms are color-coded. MFI, mean fluorescence intensity. Asterisks indicate statistically significant difference between the groups using

one way ANOVA test between the different groups for each timepoint with Benjamini-Hochberg correction (*, $P \leq 0.05$; **, $P \leq 5E-5$; ***, $P \leq 5E-10$; ****, $P \leq 5E-15$)

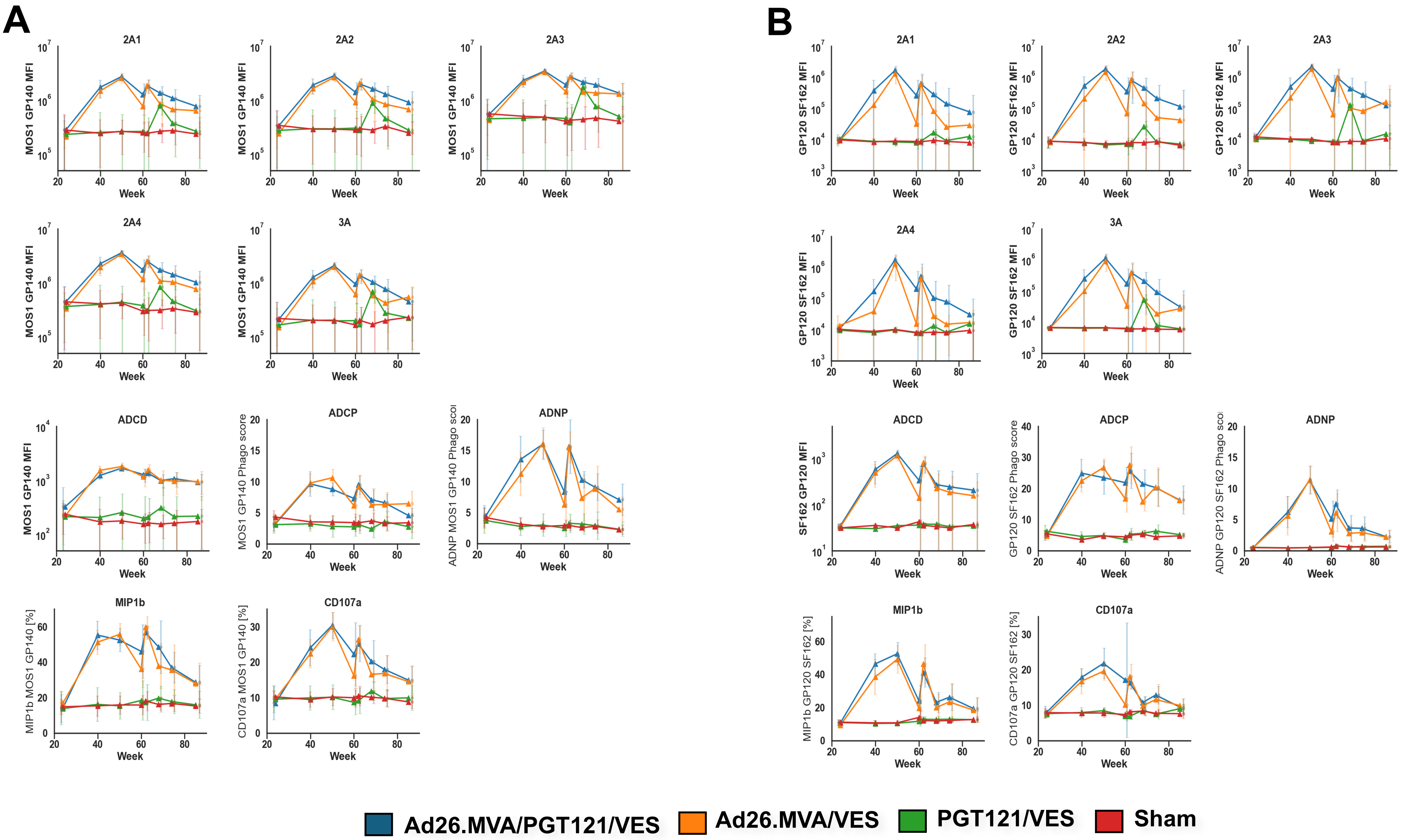
A
B
2A1
2A2
2A3
2A4
3A
ADCD
ADCP
ADNP
MIP1b
CD107a
MOS1 GP140 MFI
GP120 SF162 MFI
SF162 GP120 MFI
MOS1 GP140 Phago score
ADNP MOS1 GP140 Phago score
GP120 SF162 Phago score
ADNP GP120 SF162 Phago score
MIP1b MOS1 GP140 [%]
CD107a MOS1 GP140 [%]
MIP1b GP120 SF162 [%]
CD107a GP120 SF162 [%]
Week
Ad26.MVA/PGT121/VES
Ad26.MVA/VES
PGT121/VES
Sham

Supplementary Figure 4: Kinetics of Mos1 gp140 and SF162 gp120-specific antibody levels following therapeutic vaccination in the NHP study. Levels of Mos1 gp140 (A) and SF162 gp120 (B) -specific Fcγ-R binding and antibody functions throughout the study. The different study arms are color-coded. MFI, mean fluorescence intensity.

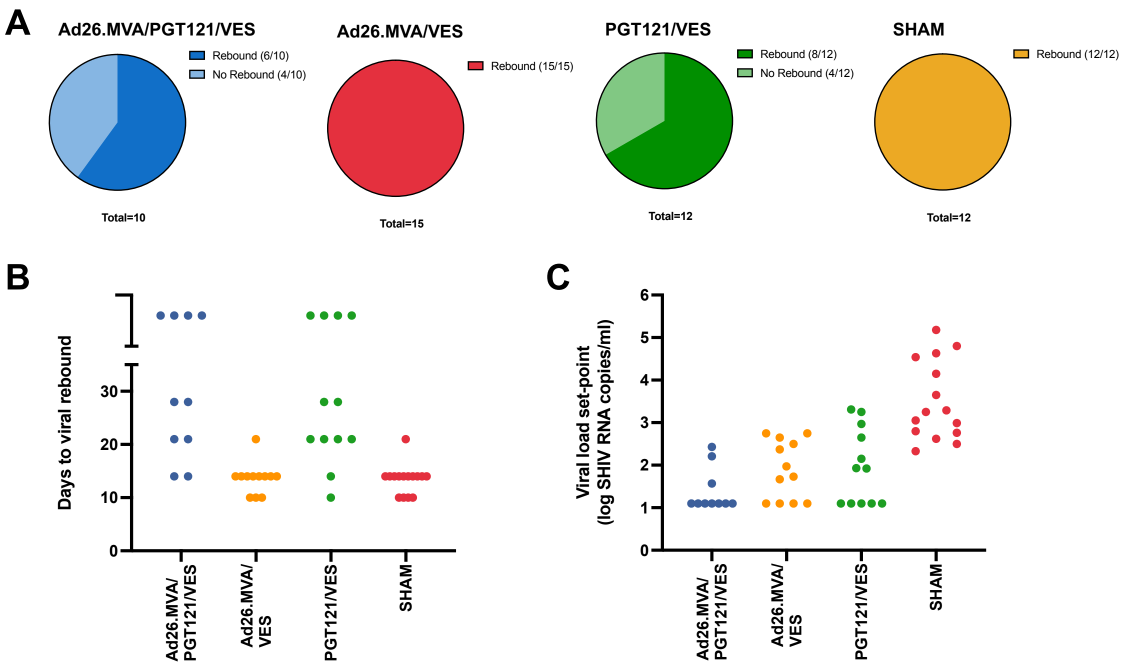

Supplementary Figure 5: Virological outcomes of the NHP study as per Walker-Sperling et al (26). (A) The number of animals in each group that rebounded following cessation of ART to the end of the surveillance period at day 168. (B) The dot-plot visualizes the days to rebound following cessation of ART. (C) Viral load set points (Log SHIV RNA copies/ml) following cessation of ART.

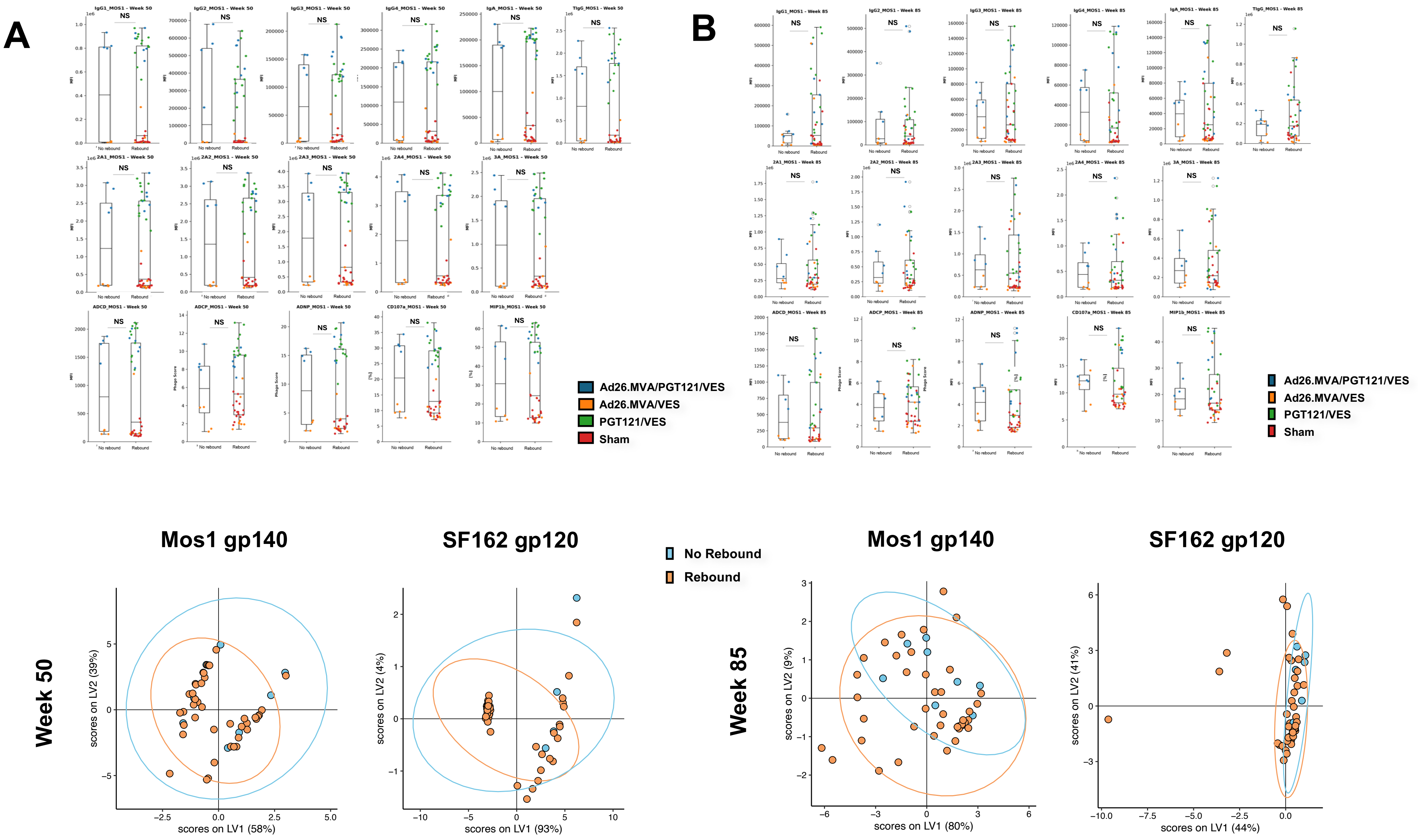
A
IgG1_MOS1 - Week 50
IgG2_MOS1 - Week 50
IgG3_MOS1 - Week 50
IgG4_MOS1 - Week 50
IgA_MOS1 - Week 50
TIgG_MOS1 - Week 50
2A1_MOS1 - Week 50
2A2_MOS1 - Week 50
2A3_MOS1 - Week 50
2A4_MOS1 - Week 50
3A_MOS1 - Week 50
ADCD_MOS1 - Week 50
ADCP_MOS1 - Week 50
ADNP_MOS1 - Week 50
CD107a_MOS1 - Week 50
MIP1b_MOS1 - Week 50
NS
No rebound
Rebound
Ad26.MVA/PGT121/VES
Ad26.MVA/VES
PGT121/VES
Sham
B
IgG1_MOS1 - Week 85
IgG2_MOS1 - Week 85
IgG3_MOS1 - Week 85
IgG4_MOS1 - Week 85
IgA_MOS1 - Week 85
TIgG_MOS1 - Week 85
2A1_MOS1 - Week 85
2A2_MOS1 - Week 85
2A3_MOS1 - Week 85
2A4_MOS1 - Week 85
3A_MOS1 - Week 85
ADCD_MOS1 - Week 85
ADCP_MOS1 - Week 85
ADNP_MOS1 - Week 85
CD107a_MOS1 - Week 85
MIP1b_MOS1 - Week 85
Ad26.MVA/PGT121/VES
Ad26.MVA/VES
PGT121/VES
Sham
Week 50
Mos1 gp140
SF162 gp120
scores on LV1 (58%)
scores on LV2 (39%)
scores on LV1 (93%)
scores on LV2 (4%)
No Rebound
Rebound
Week 85
Mos1 gp140
SF162 gp120
scores on LV1 (80%)
scores on LV2 (9%)
scores on LV1 (44%)
scores on LV2 (41%)

Supplementary Figure 6: Features associated with rebound. (A-B) Bar plots comparing Mos-1 Env-specific features in rebound and non-rebound group at week 50 (A) and week 85 (B). Data were analyzed using the Mann–Whitney U test. Values are shown as medians with interquartile ranges (IQR); whiskers represent 1.5× the IQR. (C) Score plot from PLSDA model built with SF162- and Mos-1-specific features at week 50 and 85. Each dot represents a sample, with a different color indicating whether it is in the rebound or no-rebound group. The ellipses represent 95% confidence intervals for each group. LV1: latent variable 1. LV2: latent variable 2.

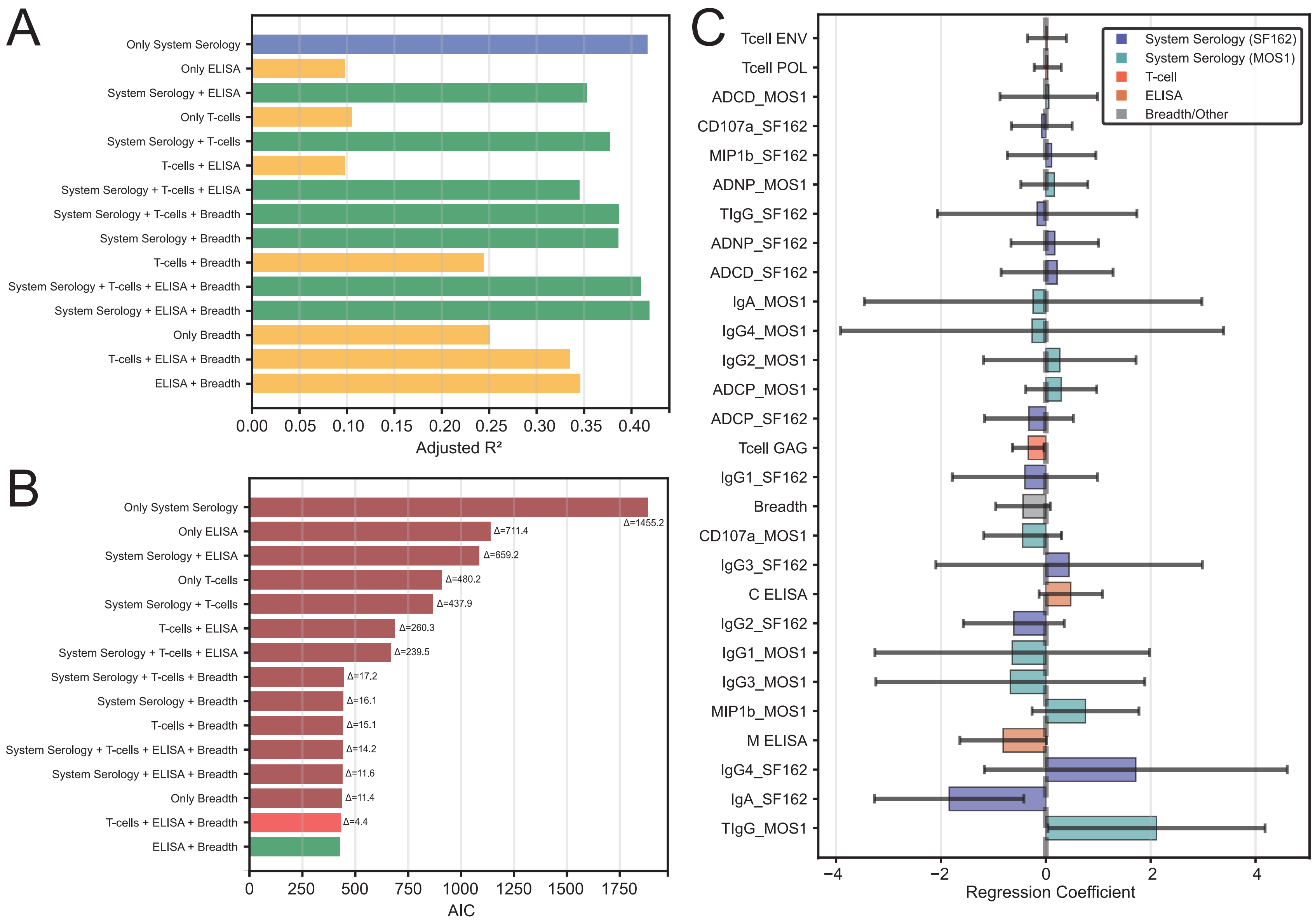

A
Only System Serology
Only ELISA
System Serology + ELISA
Only T-cells
System Serology + T-cells
T-cells + ELISA
System Serology + T-cells + ELISA
System Serology + T-cells + Breadth
System Serology + Breadth
T-cells + Breadth
System Serology + T-cells + ELISA + Breadth
System Serology + ELISA + Breadth
Only Breadth
T-cells + ELISA + Breadth
ELISA + Breadth
0.00 0.05 0.10 0.15 0.20 0.25 0.30 0.35 0.40
Adjusted R²
B
Only System Serology Δ=1455.2
Only ELISA Δ=711.4
System Serology + ELISA Δ=659.2
Only T-cells Δ=480.2
System Serology + T-cells Δ=437.9
T-cells + ELISA Δ=260.3
System Serology + T-cells + ELISA Δ=239.5
System Serology + T-cells + Breadth Δ=17.2
System Serology + Breadth Δ=16.1
T-cells + Breadth Δ=15.1
System Serology + T-cells + ELISA + Breadth Δ=14.2
System Serology + ELISA + Breadth Δ=11.6
Only Breadth Δ=11.4
T-cells + ELISA + Breadth Δ=4.4
ELISA + Breadth
0 250 500 750 1000 1250 1500 1750
AIC
C
Tcell ENV
Tcell POL
ADCD_MOS1
CD107a_SF162
MIP1b_SF162
ADNP_MOS1
TIgG_SF162
ADNP_SF162
ADCD_SF162
IgA_MOS1
IgG4_MOS1
IgG2_MOS1
ADCP_MOS1
ADCP_SF162
Tcell GAG
IgG1_SF162
Breadth
CD107a_MOS1
IgG3_SF162
C ELISA
IgG2_SF162
IgG1_MOS1
IgG3_MOS1
MIP1b_MOS1
M ELISA
IgG4_SF162
IgA_SF162
TIgG_MOS1
System Serology (SF162)
System Serology (MOS1)
T-cell
ELISA
Breadth/Other
−4 −2 0 2 4
Regression Coefficient

Supplementary Figure 7: Ordinary Least Squares regression models performance. (A) Adjusted R^2 for all evaluated models. Models are color-coded: blue (Only System Serology), yellow (models excluding System Serology), and green (models combining System Serology with T-cell breadth, magnitude and/or binding antibodies by ELISA). (B) Akaike Information Criterion (AIC) for all models, sorted from highest to lowest AIC; Δ values indicate the difference relative to the best-fitting model (lowest AIC). (C) Regression coefficients obtained from the full model that includes System Serology, T-cell breadth, magnitude and/or binding antibodies by ELISA.

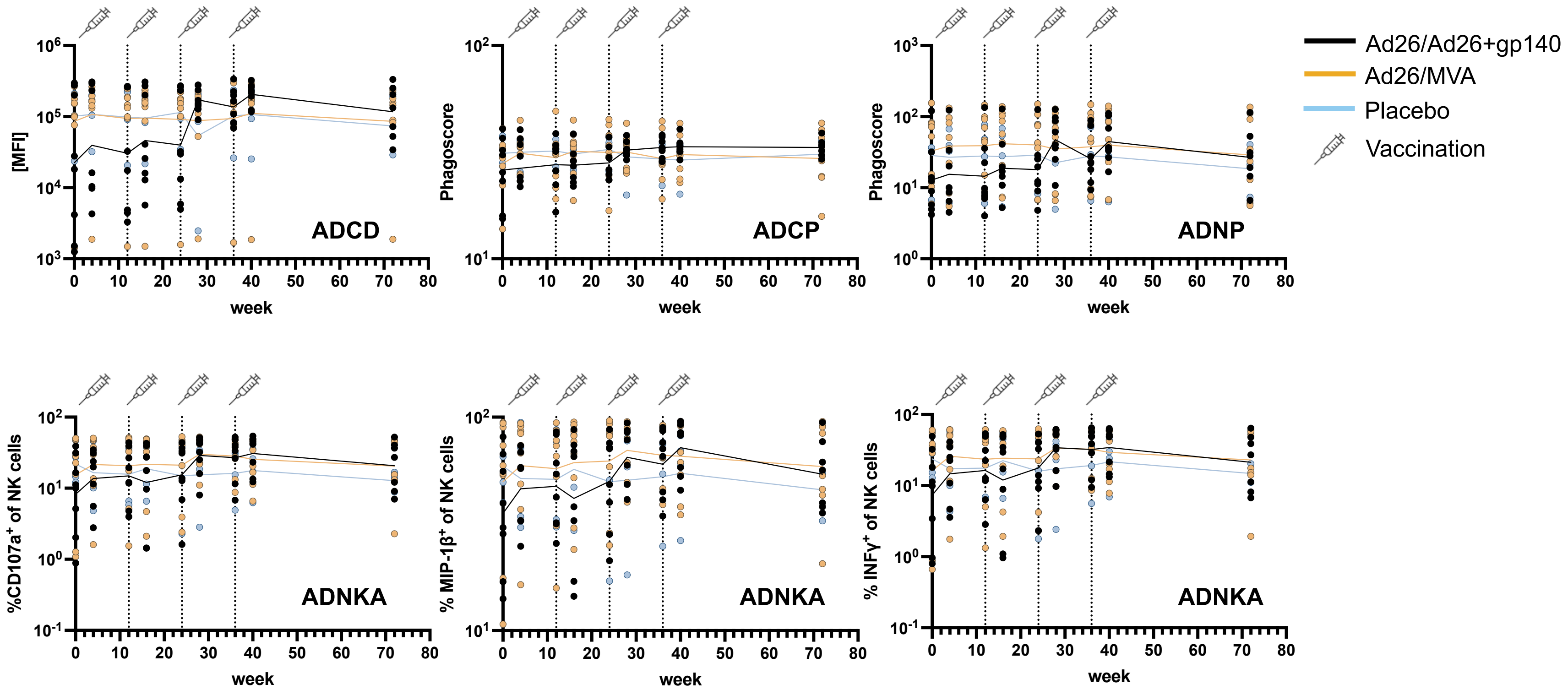

Supplementary Figure 8: Kinetics of functional antibody response following therapeutic vaccination in the HTX1002 study. Raw data of functional response levels for Mos1 Env-specific ADCD, ADCP, ADNP and ADNKA (as determined by CD107a expression or Mip-1β or IFNγ secretion) throughout the study. The different study arms are color-coded. Data is shown as geometric means. The vertical dotted lines represent the timepoints of vaccine/placebo administration.

Fcγ-Receptor	antigen	R^2
2A1	GP120 SF162	0.958557
	MOS1 GP140	0.934697
2A2	GP120 SF162	0.928011
	MOS1 GP140	0.923655
2A3	GP120 SF162	0.947708
	MOS1 GP140	0.91134
2A4	GP120 SF162	0.983786
	MOS1 GP140	0.95252
3A	GP120 SF162	0.958889
	MOS1 GP140	0.896766

Supplementary Table 1: Spearman correlation (R^2) of Fcγ-R binding antibody levels specific to the two antigens, SF162 gp120 and Mos-1 gp140, between animals that received the vaccine alone and those that received the vaccine plus PGT121.
